# Epigenetic silencing of LDHB promotes hepatocellular carcinoma by remodeling the tumor microenvironment

**DOI:** 10.1007/s00262-024-03717-2

**Published:** 2024-05-13

**Authors:** Peng Zhang, Yi Wan, Jinrong Ma, Jin Gong, Ziwei Zhong, Yuxin Cui, Hongli Zhang, Yanyan Da, Junpeng Ma, Chenxi Li, Lijuan Liu, Tian Gong, Youwen Tan, Chengsheng Zhang

**Affiliations:** 1 Center for Molecular Diagnosis and Precision Medicine, The First Affiliated Hospital, Jiangxi Medical College, Nanchang University, 1519 Dongyue Dadao, Nanchang, 330209 China; 2 Department of Clinical Laboratory, The First Affiliated Hospital, Jiangxi Medical College, Nanchang University, 17 Yongwai Zhengjie, Nanchang, 330006 China; 3 Jiangxi Provincial Center for Advanced Diagnostic Technology and Precision Medicine, The First Affiliated Hospital, Jiangxi Medical College, Nanchang University, 1519 Dongyue Dadao, Nanchang, 330209 China; 4 Jiangxi Medical Academy of Nutrition and Health Management, The First Affiliated Hospital, Jiangxi Medical College, Nanchang University, 17 Yongwai Zhengjie, Nanchang, 330006 China; 5 Cancer Center, The First Affiliated Hospital of Xi’an Jiaotong University, 277 Yanta West Road, Xi’an, 710061 China; 6 Precision Medicine Center, The First Affiliated Hospital of Xi’an Jiaotong University, 277 Yanta West Road, Xi’an, 710061 China; 7 Department of Medical Genetics, The First Affiliated Hospital, Jiangxi Medical College, Nanchang University, 1519 Dongyue Dadao, Nanchang, 330209 China

**Keywords:** Hepatocellular carcinoma (HCC), DNA methylation, DNMT3A, LDHB, Tumor microenvironment

## Abstract

Lactate dehydrogenase B (LDHB) reversibly catalyzes the conversion of pyruvate to lactate or lactate to pyruvate and expressed in various malignancies. However, the role of LDHB in modulating immune responses against hepatocellular carcinoma (HCC) remains largely unknown. Here, we found that down-regulation of lactate dehydrogenase B (LDHB) was coupled with the promoter hypermethylation and knocking down the DNA methyltransferase 3A (DNMT 3A) restored LDHB expression levels in HCC cell lines. Bioinformatics analysis of the HCC cohort from The Cancer Genome Atlas revealed a significant positive correlation between LDHB expression and immune regulatory signaling pathways and immune cell infiltrations. Moreover, immune checkpoint inhibitors (ICIs) have shown considerable promise for HCC treatment and patients with higher LDHB expression responded better to ICIs. Finally, we found that overexpression of LDHB suppressed HCC growth in immunocompetent but not in immunodeficient mice, suggesting that the host immune system was involved in the LDHB-medicated tumor suppression. Our findings indicate that DNMT3A-mediated epigenetic silencing of LDHB may contribute to HCC progression through remodeling the tumor immune microenvironment, and LDHB may become a potential prognostic biomarker and therapeutic target for HCC immunotherapy.

**Supplementary Information:**

The online version contains supplementary material available at 10.1007/s00262-024-03717-2.

## Introduction

Hepatocellular carcinoma (HCC)is ranked as the sixth most common cancer and the third leading cause of cancer death, and has become a significant global threat to human health [1]. Approximately a third of HCC patients were diagnosed in the early stages and were eligible for potentially curative treatments such as liver transplants, surgical resection, and radiofrequency ablation [2]. However, more than 50% of HCC patients are diagnosed at advanced stages, and systematic therapy is the mainstay of treatment for advanced HCC patients (such as sorafenib and Lenvatinib) [3]. Recently, immunotherapy has shown promising results for advanced HCC patients [4], and the atezolizumab combined with the bevacizumab has become the first-line treatment option for HCC patients [5, 6]. While approximately 30% of early-stage HCC patients bear evidence of immune activation, 25% have no immune infiltration [7, 8]. Therefore, understanding the underlying mechanisms responsible for the poor intratumoral immune cell infiltration will be crucial for the identification of novel biomarkers and the development of effective therapeutic strategies for HCC.

Glycolytic metabolism (also termed as the Warburg effect), which leads to the accumulation of lactate largely within the tumor microenvironment (TME), is one of the major hallmarks of cancer cells [9, 10]. A number of reports have shown that lactate in the TME can modulate immune responses, such as promoting T cell apoptosis and increasing Treg cells activity [11]. Moreover, a recent study has demonstrated that inhibition of lactate release by blocking monocarboxylate transporter (MCT) 4 promoted anti-tumor immunity of CD8^+^ T cells in HCC [12].

Lactate dehydrogenase (LDH) is a terminal enzyme in anaerobic glycolysis. LDH consists of two isoforms, LDHA and LDHB, which assemble in a tissue-specific way to form homotetramers or heterotetramers in five different combinations [13]. LDHA preferably catalyzes pyruvate into lactate, whereas LDHB predominantly reduces lactate to pyruvate. At the same time, numerous studies have demonstrated that LDHA is up-regulated in multiple cancers and plays a crucial role in tumor progression [14–16]. On the other hand, LDHB was down-regulated in several types of tumors, including liver cancer [17–19]. Moreover, low level of LDHB expression was associated with enhanced cancer cell glycolysis and lactate release as well as poor prognosis of HCC patients [19, 20]. Interestingly, decreased LDHB has recently been found to be involved in tumor immune regulation in breast cancer [21]. However, the exact roles and underlying mechanisms of low expression LDHB in HCC are still unknown.

In this study, we comprehensively evaluated the role of LDHB in HCC through bioinformatics analysis and experimental studies. We examined the expression level of LDHB in human HCC tissues, cell lines, and mouse HCC cell lines, respectively, and observed that HCC patients with down-regulated LDHB had a worse prognosis. We also investigated the epigenetic mechanisms of silenced LDHB expression and uncovered the DNMT3A-mediated aberrant methylation of the LDHB promoter in HCC cell lines. Moreover, enrichment analysis was conducted to identify the biological roles of LDHB in HCC. We noticed that LDHB could remodel the immune microenvironment of HCC and affect the immunotherapy response. Furthermore, we found that overexpression of LDHB suppressed HCC growth in immunocompetent but not in immunodeficient mice. Our findings suggest that LDHB may act as a novel suppressor of HCC and modulate TME by regulating the infiltration of immune cells.

## Materials and methods

### Data collection

The clinical information and RNA sequencing data were obtained from The Cancer Genome Atlas (TCGA, https://www.cancer.gov/tcga) database (tumor type: LIHC; tumor cases:374; normal cases:50) and Gene Expression Omnibus (GEO, https://www.ncbi.nlm.nih.gov/geo/). HTseq-TPM was the respective workflow type utilized for TCGA-LIHC. The gene dependencies of liver cancer cell lines were obtained from the DepMap website (https://depmap.org/portal/download/) using CRISPR (DepMap Public 23Q4 + Score, Chronos) and RNAi (Achilles + DRIVE + Marcotte, DEMETER2) datasets.

### Identification of differentially expressed genes and enrichment analysis

Differentially expressed genes (DEGs) in the TCGA database were analyzed using the limma R package with |log2FC|> 1.0 and false discovery rate (FDR) < 0.05 as the cut-off value. ClueGO [22] was a cytoscape software-based visualization tool used for the functional enrichment analysis of the DEGs. Gene Ontology (GO) and Kyoto Encyclopedia of Genes and Genomes (KEGG) terms were enriched through the clusterProfiler R package, and visual analysis of data was performed by ggplot R package. The Mantel test was used to analyze the correlation between LDHB and the hallmark gene sets.

### Immune-related analysis

The XCELL, MCPCOUNTER, CIBERSORT, TIMER, EPIC and QUANTISEQ algorithms were utilized for analysis of immune microenvironment. The ESTIMATE R package was utilized to study the association between the infiltration proportion of immune cells and LDHB expression. The limma R package was utilized to analyze the differential expression of immune checkpoint inhibitors related genes between the high and low expression groups of LDHB. The Immunophenoscore (IPS), a machine learning algorithm from The Cancer Immunome Database (TCIA, https://tcia.at/home), was utilized to quantify the IPS score of patients based on their transcriptional profile, indicating the response to immunotherapy.

### Cell culture and reagents

293T, PLC, HepG2 and Hepa1-6 cells were obtained from American Type Culture Collection (ATCC) and cultured in Dulbecco’s modified Eagle’s medium (Hyclone) supplemented with 10% fetal bovine serum (FBS, Zeta Life), 100 units/ml penicillin and 100 μg/ml streptomycin (Beyotime Bio-technology). Hep3B was cultured in minimal essential medium (Procell). All cells were cultured at 37 °C in a humidified incubator with 5% CO_2_. Polyethylen imine (PEI, Polysciences), 5­Aza­CdR, Puromycin (Selleck), Lipo8000, polybrene (Beyotime Bio-technology) were obtained from the companies listed above.

### Plasmids and establishment of stable cell lines

The PLKO vectors contain the shRNAs against DNMT1 (TRCN0000021890), DNMT3A (TRCN0000035757, TRCN0000035758), and DNMT3B (TRCN0000035684), respectively, were obtained commercially (Sigma-Aldrich). LDHB CDS was subcloned into the pSin-3 × Flag empty vector as described previously [23]. These plasmids along with the helper plasmids (pMD2.G and psPAX2) were co-transfected into 293 T cells in the presence of PEI. To establish the stable cell lines, Hep3B, HepG2 or Hepa1-6 cells were infected by the lentiviruses with polybrene (10 μg /ml) and then selected with (3 μg/mL) puromycin for one to two weeks.

### RNA isolation and quantitative real-time PCR (qRT-PCR)

RNA was extracted using the Trizol reagent according to the manufacturer’s instructions, and complementary DNA (cDNA) was reversed transcribed from RNA with the HiScript II Q RT SuperMix for qPCR kit (Vazyme, China). qRT-PCR was performed using ChamQ Universal SYBR Green Master Mix (Vazyme, China) on QuantStudioTM Dx system (Life Technologies) and the relative expression levels of the mRNA were calculated by 2^−ΔΔCt^ method and normalized to β-actin. The primers used for qRT-PCR are listed below:: human LDHB F: 5’- CCTCAGATCGTCAAGTACAGTCC-3’; human LDHB R: 5’-ATCACGCGGTGTTTGGGTAAT-3’; mouse LDHB F: 5’-CATTGCGTCCGTTGCAGATG-3’; mouse LDHB R: 5’- GGAGGAACAAGCTCCCGTG-3’; DNMT1 F: 5’- CCTAGCCCCAGGATTACAAGG-3’; DNMT1 R: 5’-ACTCATCCGATTTGGCTCTTTC-3’; DNMT3A F: 5’-CCGATGCTGGGGACAAGAAT-3’; DNMT3A R: 5’-CCCGTCATCCACCAAGACAC-3’; DNMT3B F: 5’-CCCAGCTCTTACCTTACCATCG-3’; DNMT3B R:5’- GGTCCCCTATTCCAAACTCCT-3’. Experiments were performed at least twice.

### Bisulfite DNA sequencing

Bisulfite DNA sequence was performed as described previously [24]. Briefly, DNA was bisulfite modified following the protocol of the QIAGEN kit (Cat. # 59824). Nest PCR was used to amplify bisulfite-treated DNA using the primers described below: LDHB-P1-F: 5’- AGTTTTTTAAAGTTTAATTGAAATGT-3’; LDHB-P1-R: 5’- ATTTTAAAACRAAATCTCACTCT-3’; LDHB-P2-F: 5’-GGTTTATAGGTAAGTTTGATGGG-3’; LDHB-P2-R: 5’-AAATACAACAAATCTCTCCRCTA-3’. LDHB promoter sequences from the amplified bisulfite-treated DNA were compared to those from the original DNA.

### Cell proliferation and colony formation

For cell proliferation assay, 5 × 10^3^ cells were seeded in 96-well plates, and cell numbers were counted using Cell Counting Kit-8 (CCK-8, Cat# C0005, TargetMol, USA) reagents every day over a 4-day period. For colony formation, 1 × 10^4^ cells were seeded in six-well plates and were stained with 0.2% crystal violet containing 4% PFA after 7–14 days. Experiments were performed at least twice.

### Western blot

The cells were lysed with RIPA (Beyotime) supplemented with protease inhibitor cocktail (Beyotime) and lysates containing equal amounts of protein were boiled and fractionated by 6–10% SDS-PAGE. The primary antibodies included LDHB (11,204–1-AP, Proteintech, PTM-5869, PTM BIO), LDHA (19,987–1-AP, Proteintech), Flag (15,938–1-AP, Proteintech), and β-actin (66,009–1-lg, Proteintech). The signals were detected with Western ECL Substrate (Bio-Rad) using HRP-conjugated anti-rabbit and anti-mouse secondary antibodies (Abclonal) and visualized using the AMERSHAM Imagequant 800 imaging System. Experiments were performed at least twice.

### In vivo experiments

All experimental procedures involving mice were approved by the Ethics Committee of the First Affiliated Hospital of Nanchang University. The male BALB/c nude mice and male C57BL/6 mice (GemPharmatech) were housed under specific pathogen-free conditions. Mouse hepatoma cell line Hepa1-6 cells stably overexpressing LDHB and control cells were injected subcutaneously into the right posterior flanks of 6-week-old BALB/c nude male mice (3 × 10^6^ cells) or the right forelimbs flanks of 6-week-old C57BL/6 male mice (1 × 10^7^ cells). Tumor growth was assessed using standard caliper measurement every 2–3 days and calculated using the following formula: length (mm) × width (mm) × height (mm) × 0.52. After 20–25 days of treatment, mice were sacrificed and tumor tissues were isolated, and their weights and volumes were measured at the experimental endpoint. Tumor volumes and weights were analyzed using a paired Student’s t-test.

### Immunohistochemistry (IHC)

The tissue microarrays of human HCC were purchased from Bioaitech (Xian, China). The tissues were stained with anti-LDHB antibody and IHC chromogen intensity was quantified as previously described [25]. Briefly, the staining intensity (0, 1, 2, 3) and the percentage of positive cells among cancer (0–25% recorded as 1, 25–50% as 2, 50–75% as 3 and > 75% as 4) were determined by a pathologist, and the immunoreactive scores (IRS) = the staining intensity × the percentage of positive cells among cancer.

### Statistical analysis

The data were obtained from two or three independent experiments, and were presented as mean ± SD or mean ± SEM. Overall survival was represented with Kaplan–Meier curves. Statistical analyses were carried out using GraphPad Prism 6 software (La Jolla, CA, USA) and R software, and Student’s t-test was used to calculate *p*-values. Significant differences were considered when *p* < 0.05 was achieved.

## Results

### Decreased LDHB expression level in HCC correlated with poor prognosis

**Fig. 1:**
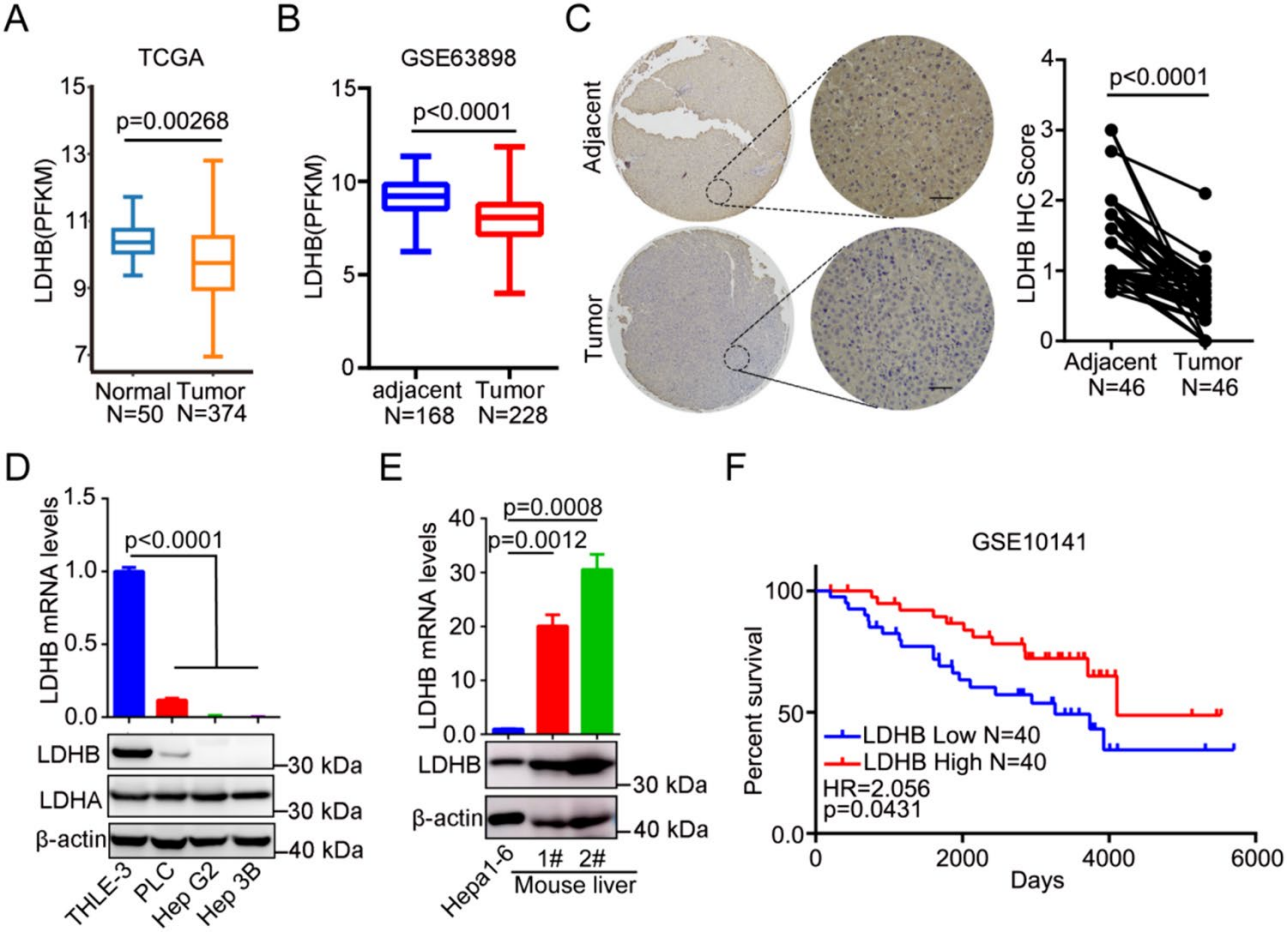
Down-regulation of LDHB in HCC tissues and cell lines, and correlation with poor overall survival in HCC patients. **A**, **B** LDHB expression levels in HCC and nontumor tissues in TCGA (Data from DNMIVD, http://119.3.41.228/dnmivd/index/) and GSE63898 databases. **C** Representative images and statistical analysis of LDHB IHC staining intensity in 46 paired HCC patient tissues. Scale bars, 25 μm. **D** LDHB expression levels were detected in human HCC cell lines PLC, HepG2, Hep3B by qRT-PCR and Western blot. The human liver epithelial cell line THLE3 was used as a control. **E** LDHB expression level was detected in mouse HCC cell line Hepa1-6 by Western blot and qRT-PCR. Mouse normal liver tissues were used as controls. **F** Kaplan–Meier OS curves for HCC patients with high or low LDHB levels in the GSE10141database

To evaluate the potential role of LDHB in HCC, we first analyzed the LDHB mRNA expression in normal (adjacent) and HCC tissues in both TCGA [26] database and GSE63898 datasets, and revealed that LDHB expression level was markedly decreased in the tumor tissues (Fig. 1A, 1B). In addition, tissue array analysis was used to evaluate the protein expression of LDHB in HCC samples. As compared to adjacent nontumor tissues, 46 cases of HCC tissue expressed significantly less LDHB protein (Fig. 1C). Moreover, we validated the expression levels of LDHB mRNA and protein in four different liver cancer cell lines by qRT-PCR and western blotting, respectively. All three human cancer cell lines showed silenced LDHB expression compared to human normal liver THLE3 cells (Fig. 1D) and mouse hepatoma Hepa1-6 cells also showed silenced LDHB expression compared to mouse normal liver tissue (Fig. 1E).

To further understand the prognostic value of LDHB expression in HCC, GSE10141 dataset was used to assess the survival prognosis. Consistent with the previous study [20], we found that a significant correlation between high LDHB levels and improved overall survival (OS, *p* = 0.0431) (Fig. 1F), suggesting that high levels of LDHB in HCC tissues might be associated with a favorable prognosis in patients.

### DNMT 3A mediates hypermethylation of LDHB promoter in HCC

**Fig. 2:**
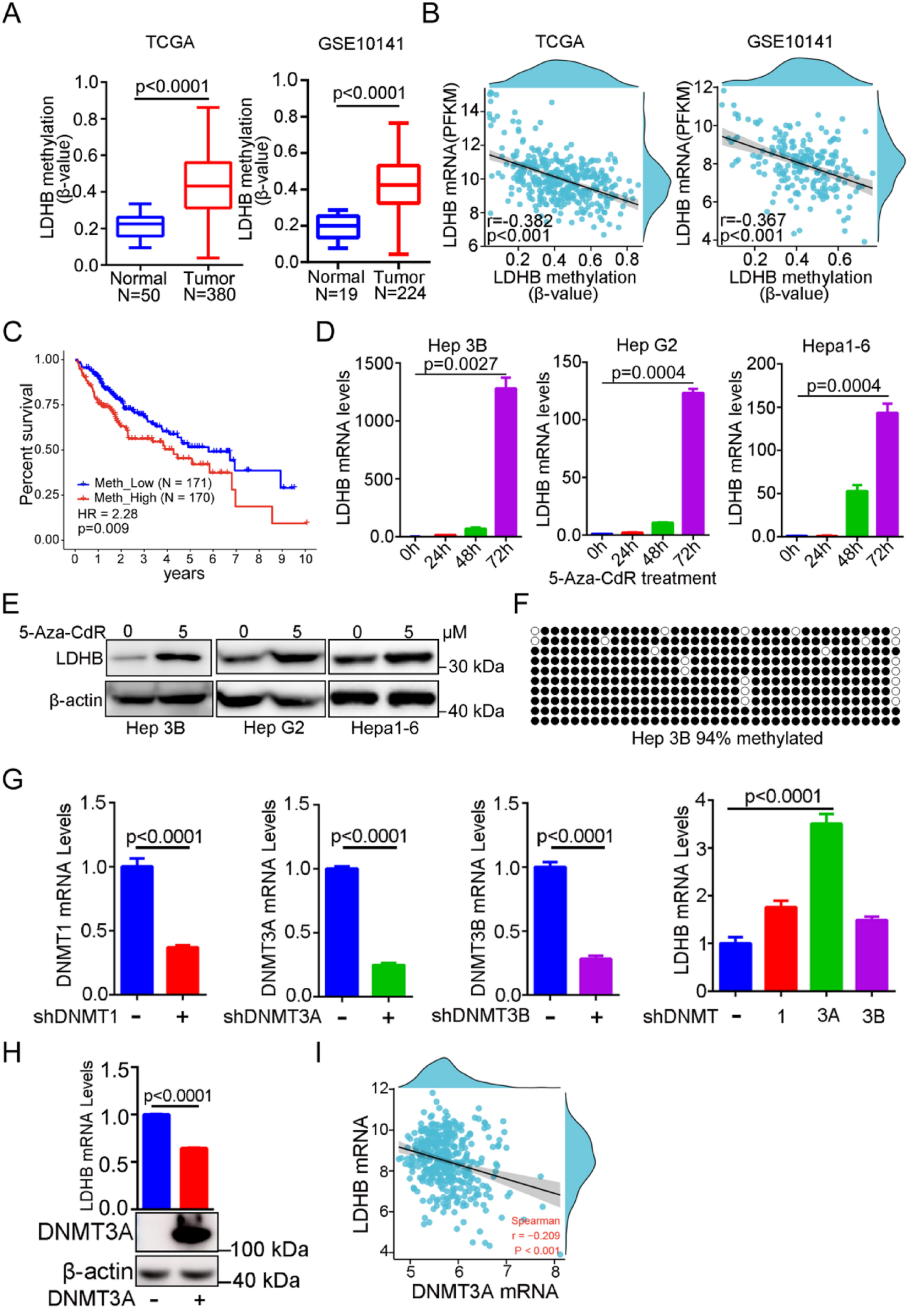
Hypermethylation of LDHB promoter in HCC tissues and cell lines. **A** Box plots showing the LDHB promoter methylation status in normal liver and tumor specimens from TCGA and GSE10141 databases. **B** LDHB mRNA expression in tumor tissues was inversely correlated with the level of LDHB promoter methylation in TCGA (N = 380) and GSE10141 (N = 204) databases. **C** Kaplan–Meier OS curves for HCC patients with high or low LDHB methylation levels in TCGA database. **D** qRT-PCR results showed that LDHB mRNA levels were significantly elevated when Hep3B, HepG2 and Hepa1-6 cells were treated with 5­Aza­CdR at 5 μM for the indicated times. **E** The protein level of LDHB was detected by Western blotting when Hep3B cells, HepG2 and Hepa1-6 cells were treated with 5­Aza­CdR at 5 μM for 96 h. **F** The methylation status of the LDHB promoter region was analyzed in Hep3B cells. Black circle is methylated whereas white circle is unmethylated. **G** The mRNA levels of DNMT 1, DNMT 3A, DNMT 3B and LDHB were detected in DNMT 1–silenced, DNMT 3A–silenced, or DNMT 3B–silenced Hep3B cells by using qRT-PCR. **H** The mRNA levels of LDHB were detected in DNMT3A-overexpressed 293 T cells for 72 h. **I** LDHB levels were inversely correlated with DNMT 3A expression in HCC tissues from TCGA­LIHC

Next, we explored the underlying mechanism of decreased LDHB expression in HCC. HCC tissues showed LDHB down-regulation at the mRNA level, indicating a pre-translational regulation mechanism. Therefore, we examined the mRNA levels of LDHB and DNA methylation status in HCC tissue samples from the TCGA and GEO databases. Indeed, tumor tissues had higher levels of hypermethylation of the LDHB promoter than non-tumor (Fig. 2A). Moreover, linear regression analysis showed that LDHB promoter methylation was significantly negatively correlated with mRNA expression (Fig. 2B), suggesting that DNA methylation may play a pivotal regulatory role in LDHB expression. This finding was consistent with a recent report that LDHB was hypermethylated in DNA methylation profiling of HCC [27]. Furthermore, there was a significant correlation between low LDHB methylation levels and improved overall survival (OS, *p* = 0.009) (Fig. 2C).

To validate that the LDHB expression is mainly dependent on the LDHB promoter methylation, we demethylated Hep3B, HepG2 and Hepa1-6 cells using 5­Aza­2′­deoxycytidine (5­Aza­CdR). LDHB mRNA levels were dramatically increased in all cell lines treated with 5­Aza­CdR at 5 μM in a time-dependent manner (Fig. 2D). Consistently, western blotting results also showed that Hep3B, HepG2 and Hepa1-6 cells treated with 5­Aza­CdR for 96 h had significantly elevated LDHB protein levels (Fig. 2E). We further determined the methylation levels of the LDHB promoter region using bisulfate sequencing. As a result, a 94% methylation level was observed in Hep3B cells for the LDHB promoter (Fig. 2F). Taken together, these results suggested that the hypermethylation status of the promoter results in the suppression of LDHB expression in HCC cells.

Three enzymes catalyze DNA methylation in mammals: DNA methyltransferase 1 (DNMT 1), DNMT 3A and DNMT 3B [28]. To confirm which DNA methyltransferase mediated LDHB promoter methylation, we examined LDHB levels in Hep3B cells with DNMT 1 knockdown, DNMT 3A knockdown, and DNMT 3B knockdown, respectively. qRT-PCR results showed that only DNMT 3A knockdown significantly increased the mRNA level of LDHB (Fig. 2G), suggesting that DNMT 3A induced the aberrant methylation of the LDHB promoter in HCC cells. We then determined the expression of the LDHB in DNMT 3A overexpressed 293 T cells, and found the mRNA levels of LDHB were dramatically decreased (Fig. 2H). A correlation analysis of LDHB and DNMT 3A expression using the TCGA­LIHC dataset was conducted to further evaluate their physiological significance, and found that the mRNA levels of LDHB were negatively correlated with the mRNA levels of DNMT 3A in HCC tissues (r =  − 0.209, *p* < 0.001) (Fig. 2I). Our findings suggested that the hypermethylation of LDHB promoter was regulated by DNMT 3A in HCC.

### LDHB played a wide range of biological regulatory roles in HCC

**Fig. 3:**
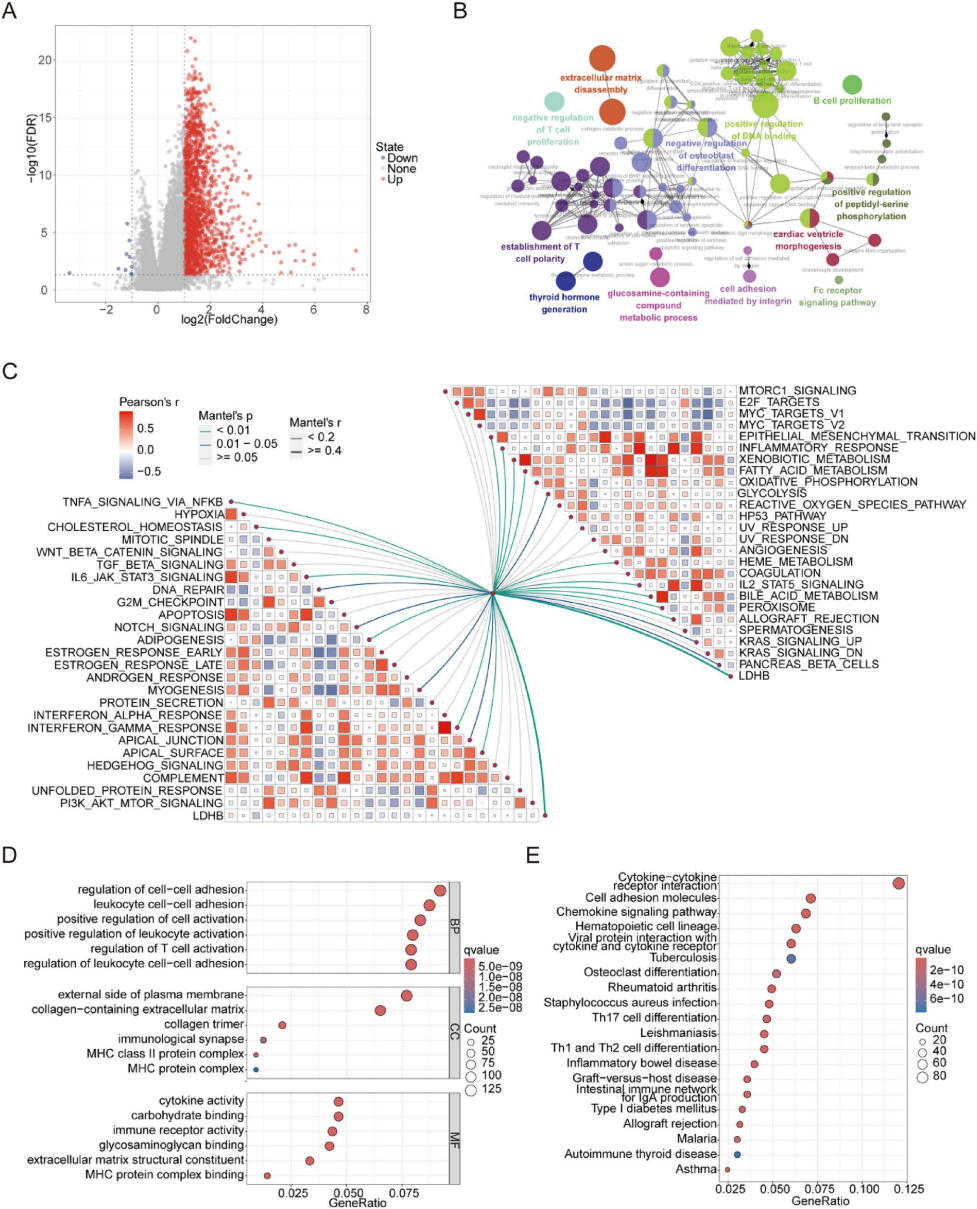
The biological roles of LDHB. **A** DEGs between the high and low LDHB expression. **B** clueGO analysis of DEGs. **C** Correlation analysis of Hallmark gene sets. **D**–**E** GO and KEGG analysis of the DEGs

To understand the biological roles of LDHB in HCC, we examined the DEGs associated with LDHB expression. A total of 10 downregulated and 1600 upregulated DEGs were identified in patients with high and low LDHB expression (Fig. 3A). These DEGs were involved in the positive regulation of DNA binding, extracellular matrix disassembly, negative regulation of osteoblast differentiation, thyroid hormone generation, B cell proliferation, negative regulation of T cell proliferation, Fc receptor signaling pathway, cell adhesion mediated by integrin, the establishment of T cell polarity, glucosamine-containing compound metabolic process, positive regulation of peptidyl-serine phosphorylation and cardiac ventricle morphogenesis based on clueGO analysis (Fig. 3B). Moreover, the correlation analysis of hallmark gene sets indicated that LDHB was positively correlated with immune regulatory pathways, such as TNFα signaling via NFKB, IL6, JAK, STAT3 signaling, interferon gamma (IFN‐γ) response (Fig. 3C). GO analysis indicated that LDHB was positively correlated with most immune terms. For example, for the Biological Process (BP), Cellular Component (CC) and Molecular Function (MF), the term was enriched in regulation of T cell activation, MHC protein complex, and immune receptor activity, respectively (Fig. 3D). For the GSEA analysis, the top enriched terms were cytokine-cytokine receptor interaction, cell adhesion molecules, chemokine signaling pathway (Fig. 3E). These results suggested that LDHB was involved in immune regulation of HCC.

### LDHB may remodel the immune microenvironment in HCC

**Fig. 4:**
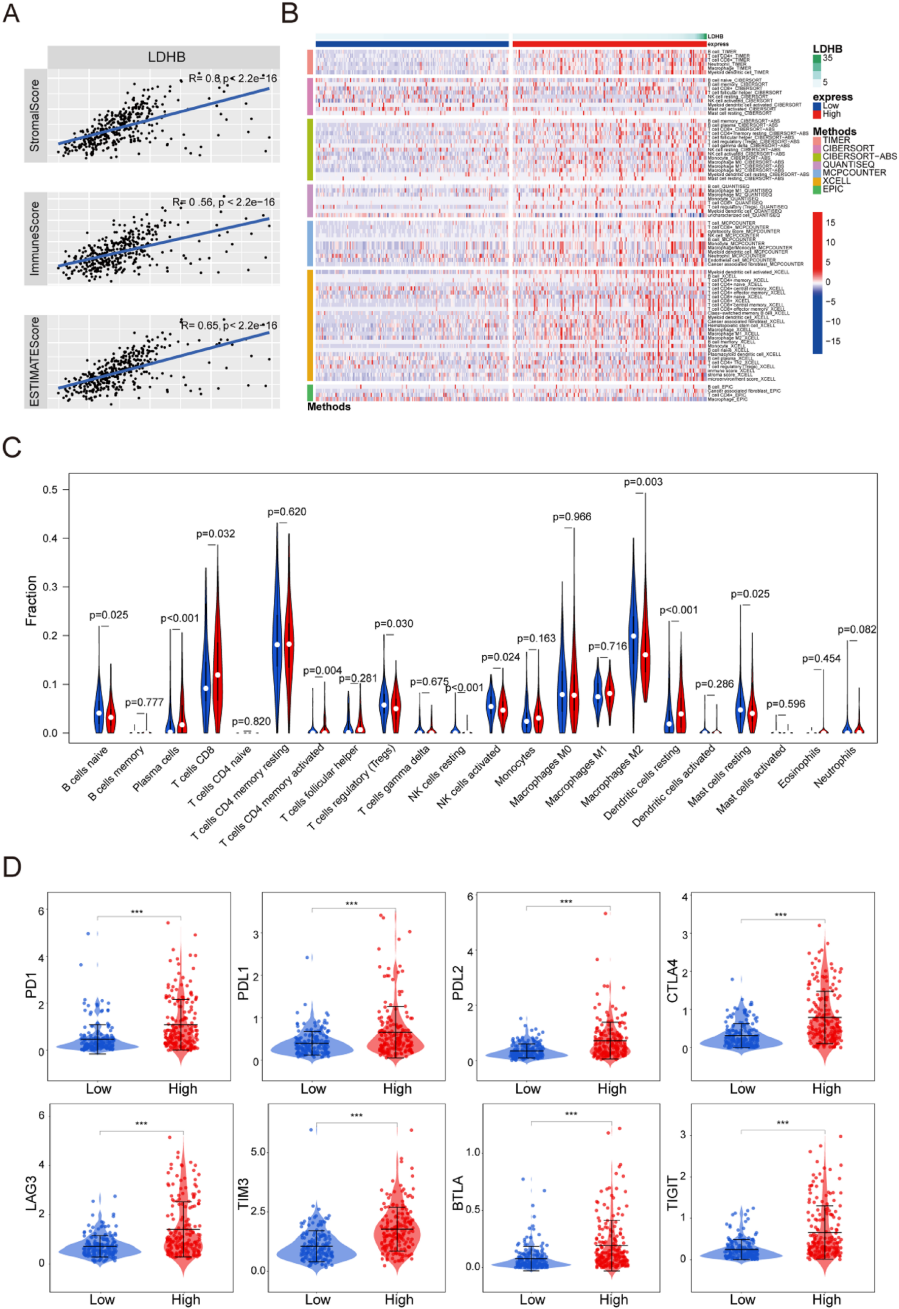
The immune landscape associated with LDHB expression levels. **A** Correlation of LDHB and immune score, stromal score and estimate score. **B** Immune infiltration of LDHB. **C** Difference analysis of TIICs between LDHB high and low expression groups. **D** The expression level of key immune checkpoints in patients with high and low LDHB expression

To further investigate the potential roles of LDHB in modulating the immune responses, we first analyzed the correlation between LDHB expression and immune cell infiltration. A significant positive correlation was observed between LDHB gene expression and immune score/ stromal score/ estimate score, using the ESTIMATE algorithm from the TCGA database (Fig. 4A). Based on the XCELL, MCPCOUNTER, CIBERSORT, TIMER, EPIC and QUANTISEQ algorithms, we quantified the immune microenvironment of HCC samples. A different immune infiltration pattern was observed in patients with high and low LDHB expression (Fig. 4B). Moreover, the proportions of plasma cells, CD8^+^T cells, activated memory CD4^+^T cells, and resting Dendritic cells were higher in the high expression group of LDHB. The proportions of naive B cells, Tregs, resting/activated NK cells, M2 macrophages, and resting mast cells were low in the high expression group of LDHB (Fig. 4C). Taken together, these results suggested that LDHB may regulate HCC progression by remodeling the tumor immune microenvironment.

Intriguingly, the expression of genes associated with T cell-exhaustion, including programmed cell death 1 (PDCD1, encoding PD-1, *p* < 0.001), and cytotoxic T lymphocyte-associated antigen-4 (CTLA-4, *p* < 0.001), Lymphocyte-activation gene 3 (LAG-3, *p* < 0.001), Hepatitis A Virus Cellular Receptor 2 (HAVCR2, encoding TIM-3, *p* < 0.001), B and T lymphocyte attenuator (BTLA, *p* < 0.001) and T-cell immunoreceptor with immunoglobulin and ITIM domain (TIGIT, *p* < 0.001) (Fig. 4D), all presented a high level in patients with higher LDHB expression. These findings indicated that HCC patients with high LDHB expression may respond better to immune checkpoint blockade therapy.

### Immunotherapy response and drug sensitivity of LDHB in HCC

**Fig. 5:**
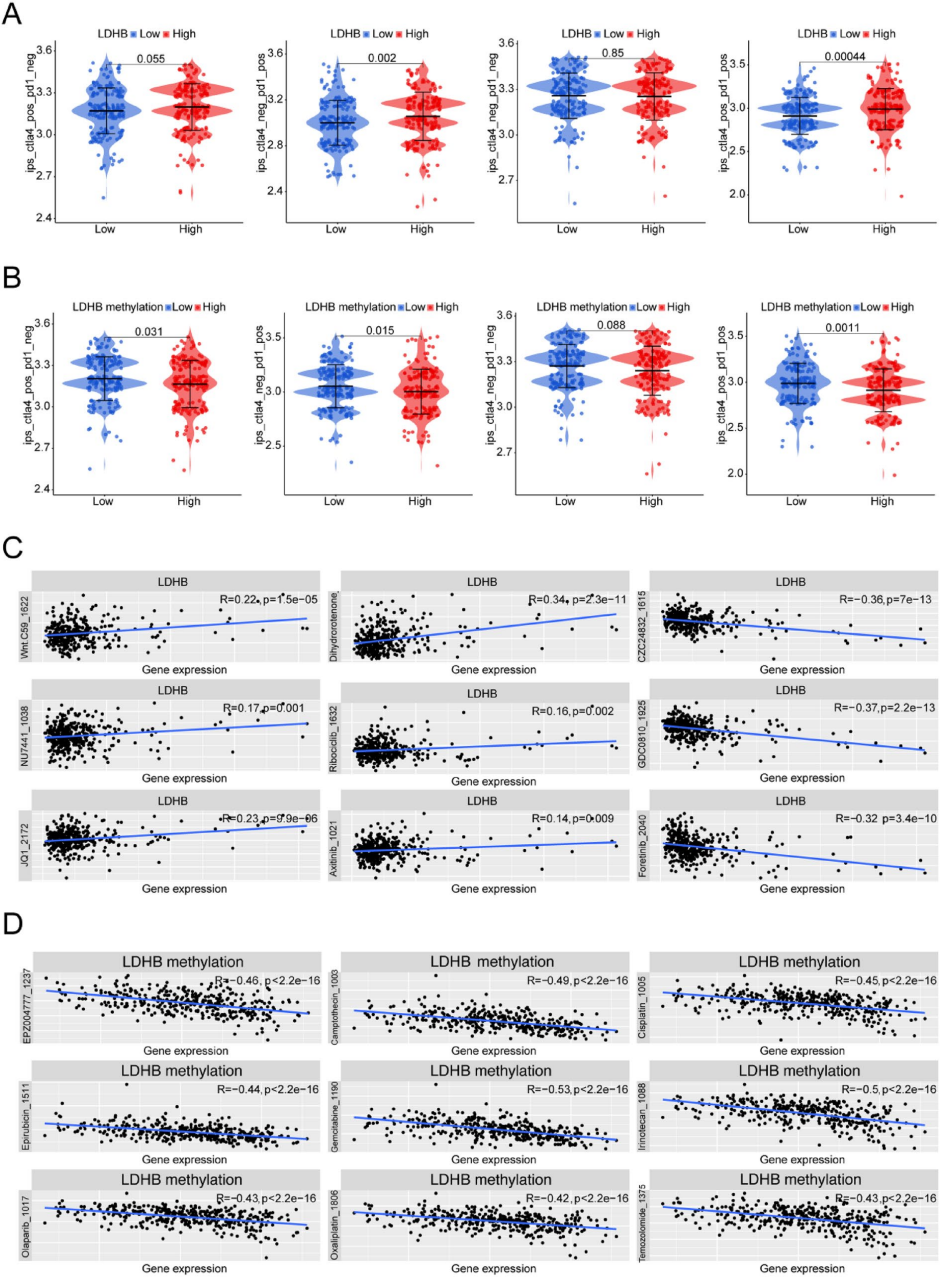
Immunotherapy and drug sensitivity associated with LDHB expression levels. **A** Difference analysis of IPS between LDHB high and low expression groups. **B** Difference analysis of IPS between LDHB high and low methylation groups. **C** Correlation of LDHB expression level and drug sensitivity. **D** Correlation of LDH methylation level and drug sensitivity

We next investigated the relationship between LDHB and immunotherapy response. We found the Immunophenoscore (IPS) (CTLA4_neg_PD1_pos and CTLA4_pos_PD1_pos) were high in the LDHB high expression group (Fig. 5A). On the other hand, the IPS (CTLA4_pos_PD1_neg, CTLA4_neg_PD1_pos and CTLA4_pos_PD1_pos) were low in the LDHB high methylation group (Fig. 5B), indicating that LDHB could affect the immunotherapy response of HCC patients. For the common chemotherapy drugs and targeted drugs, we found that LDHB methylation level could increase the sensitivity of cisplatin (R =  − 0.45, *p* < 2.2e − 16), epirubicin (R =  − 0.44, *p* < 2.2e − 16), camptothecin (R =  − 0.49, *p* < 2.2e − 16), gemcitabin (R =  − 0.53, *p* < 2.2e − 16), and irinotecan (R =  − 0.5, *p* < 2.2e − 16), compared to LDHB gene expression level (Fig. 5C and D). These findings indicated that the expression and methylation level of LDHB may serve as a biomarker for selection of therapeutic options including immune checkpoint inhibitors in HCC patients.

### Suppression of HCC progression by LDHB was dependent on the host immune system

**Fig. 6:**
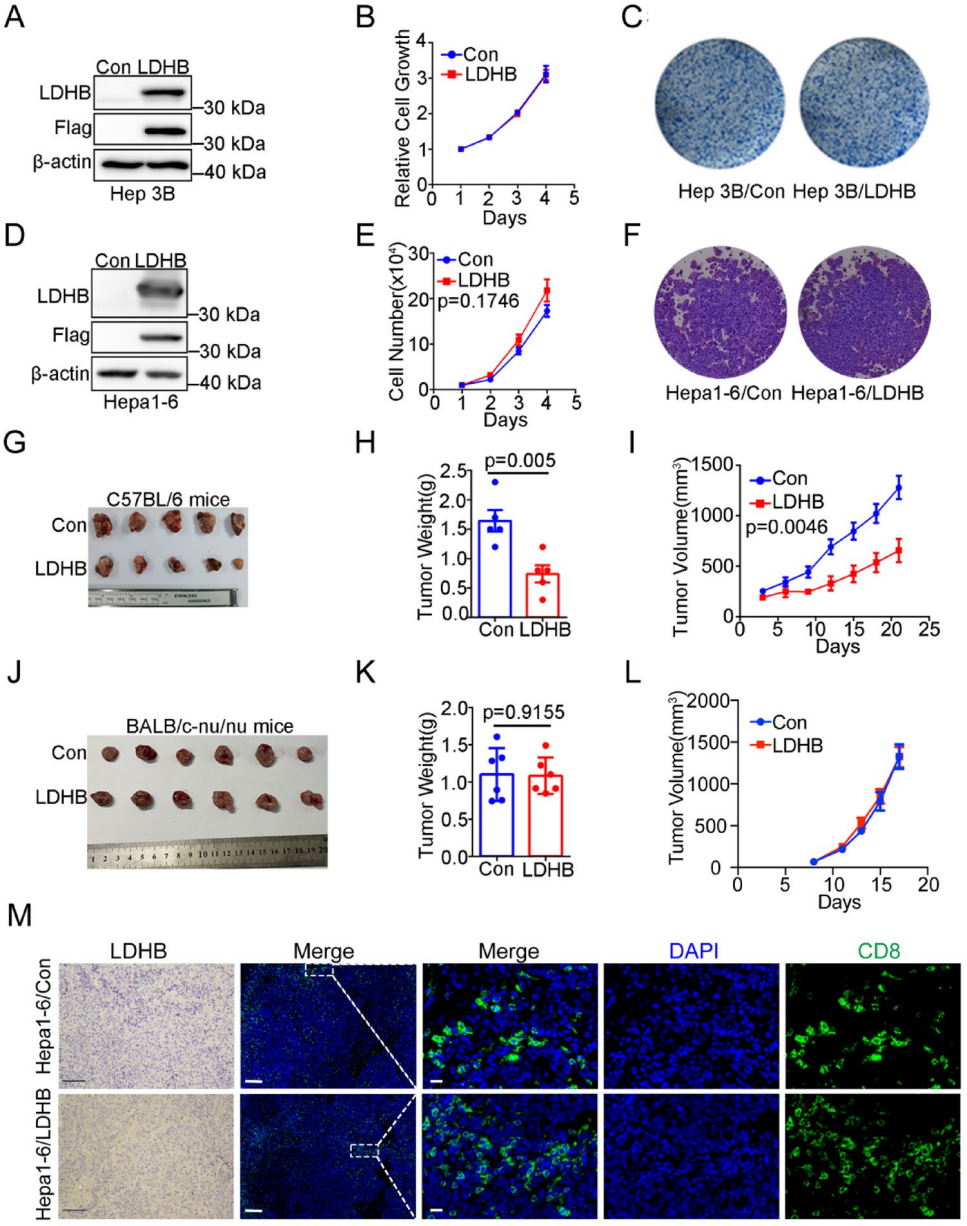
LDHB suppresses HCC progression in immune-competent mice but not in immune-deficient mice. **A** LDHB expression in Hep3B/LDHB and corresponding control cells was examined by Western blotting. **B** Cell proliferation assay was conducted with the Hep3B cells tested in A. **C** Cell colony formation assay was conducted with the Hep3B cells tested in A. **D** LDHB expression in Hepa1-6/LDHB and corresponding control cells was examined by Western blotting. **E** Cell proliferation assay was conducted with the HepA1-6 cells tested in D. **F** Cell colony formation assay was conducted with the Hepa1-6 cells tested in D. **G** Hepa1-6 cells tested in D were inoculated into C57BL/6 mice (n = 5). Homograft tumors at the endpoint were collected and shown. **H** The weight of tumors at the endpoint in G. **I** The growth curve of the tumors in G. **J** Hepa1-6 cells tested in D were inoculated into nude mice (n = 6). Xenograft tumors at the endpoint were collected and shown. **K** The weight of tumors at the endpoint in J. **L** The growth curve of the tumors in J. **M** IHC staining of LDHB expression and immunofluorescent staining of CD8^+^ T cells were performed on paraffin­embedded tumor tissues. Scale bar, 100 μm for IHC; 20 μm for immunofluorescence

Finally, we evaluated the role of LDHB in HCC in vitro and in vivo. To examine the role of LDHB in HCC in vitro, we restored LDHB expression in two HCC cell lines with silenced LDHB expression as described above. LDHB expression levels in Hep3B and Hepa1-6 cells were verified by western blotting (Fig. 6A and D). We evaluated the effect of LDHB on cell proliferation with the cell counting kit-8 (CCK-8) and the cell colony formation assay. None of the restored cell lines showed significant effect on cell proliferation compared to the control group (Fig. 6B, C, D and F). Interestingly, the CRISPR and RNAi gene dependency data from the DepMap datasets indicated that LDHB expression had no obvious relevance to the viability of HCC cell lines (Supplementary Fig. 1A, B). To further examine the role of LDHB in HCC in vivo, we subcutaneously inoculated the control mouse HCC cell line Hepa1-6/con and the Hepa1-6/LDHB into immunocompetent C57BL/6 mice, respectively. Interestingly, we observed that both the volume and weight of the tumors with stably expressed LDHB were reduced significantly in the immunocompetent mice compared to the control tumors (Fig. 6G–I). These data suggested that LDHB might require the host immune system to modulate tumor growth in vivo.

Since the host immune system plays a critical role in controlling tumor progression [29], we reasoned that LDHB loss in HCC cells might attenuate the anti-tumor immune response in vivo. To test this hypothesis, we subcutaneously inoculated the mouse HCC cell lines Hepa1­6/con and Hepa1­6/LDHB into the immunodeficient BALB/c nude mice, respectively. Indeed, LDHB expression did not affect the tumor growth in immunodeficient nude mice (Fig. 6J–L). Similar results were obtained with human liver cancer cell lines HepG2 (Supplementary Fig. 1D-E). Finally, to evaluate the relationship between the antitumor immune response and LDHB level in tumor tissues, infiltrated CD8^+^ T cells in paraffin­embedded tumor tissues were detected using immunofluorescence assay. As expected, the total CD8^+^ T cells were increased in tumors with LDHB expression compared with the control tumors in immunocompetent C57BL/6 mice (Fig. 6M), suggesting that the host immune system was required for LDHB-mediated inhibition of HCC progression.

## Discussion

In this study, we found that LDHB was down-regulated in HCC and low expression of LDHB was correlated with shorter OS in HCC patients. We also revealed that DNA methyltransferase DNMT3A could silence LDHB expression and restoring LDHB expression might suppress HCC progression through remodeling the tumor immune microenvironment. Increasing reports have shown that LDHB could be regulated by post-transcriptional modification in an FTO/m^6^A-dependent manner [30] and post-translational modifications, including acetylation [31, 32] and phosphorylation [23], but, the transcription regulation of LDHB is poorly understood. In eukaryotes, DNA methylation is one of the major manners that regulate gene expression [33]. Many researches have shown that the LDHB promoter was hypermethylated in HCC [27, 34], and other studies found that the LDHB protein level was down-regulated in HCC [19]. However, whether DNA methylation regulates LDHB expression and which methyltransferase regulates LDHB promoter hypermethylation in HCC are still unknown. We also observed that promoters of LDHB in tumor tissues were highly methylated compared to the normal tissues in the TCGA database and GSE10141 dataset. There are three methyltransferases that mediate DNA methylation modifications. We knocked down each of these methyltransferases, respectively, and found that, by only knocking down DNMT3A, the levels of LDHB mRNA were significantly increased. Therefore, we identified a novel mechanism by which the DNMT3A regulated LDHB expression at the transcriptional level by promoting methylation levels of LDHB promoters in HCC.

Currently, the roles of LDHB in tumor development are not fully understood. Some studies suggested that LDHB was essential for the proliferation of non-small cell lung cancer (NSCLC) and triple-negative breast cancer cells [35–37], whereas others reported that LDHB downregulation or loss was an important event in cancer development [13], including prostate, breast, pancreatic and liver cancers [17, 18, 27, 38], and was associated with rapid proliferation, accelerated tumor cell invasion and shorter patient survival outcomes [19, 20, 39, 40], indicating that LDHB may serve as a tumor suppressor in these cancers. Indeed, suppression of LDHB could enhance liver cancer cell glycolysis and invasiveness via lactate release in vitro [20, 40]. Recent studies demonstrated that LDHB was involved in the regulation of immune cell function. Decreased LDHB expression in breast tumor cells causes NK cell activation and promotes tumor progression [21]. LDHB regulates macrophage metabolism in breast tumor [41]. SHP1/PKM2/LDHB axis regulate glycolysis to maintain functions of T cells [42]. In addition, LDHB was involved in maintaining the antitumor activity of tumor infiltrating dendritic cells [43]. Non-alcoholic fatty liver disease (NAFLD) is one of the causes of HCC [44]. Wang et al. have shown that acetylation of LDHB drives NAFLD progression by decreasing LDHB activity and impairing lactate clearance [31]. Thus, LDHB activity is crucial for the liver to maintain normal function. However, the exact roles of LDHB in HCC progression in vivo, and whether LDHB suppresses HCC progression by affecting the tumor immune environment is unclear.

In our study, we screened stable HCC cell lines expressing LDHB and found no obvious change in cell proliferation compared with control cells in vitro. Intriguingly, there was also no difference in the growth rate of mouse HCC cell line Hepa1-6 cells expressing LDHB compared with control cells in immunodeficient BALB/c nude mice, but the growth rate of Hepa1-6 cells expressing LDHB was significantly inhibited in immunocompetent C57BL/6 mice. These results suggested that LDHB-mediated antitumor activity might be related to immune system modulation. Bioinformatics analysis of the HCC cohort from TCGA revealed that LDHB expression correlated significantly with multiple immune regulatory signaling pathways and immune cells infiltration. Notably, CD8^+^T cells were significantly enriched in HCC patients with high LDHB expression (*p* = 0.032). Consistent with our results, BALB/c nude mice were immunodeficient in T cells due to the lack of a thymus, where T cells differentiate, develop and mature. Moreover, it was reported that suppressing lactate release by blocking MCT 4 could increase CD8^+^ T cell involvement and activity in HCC [12]. In addition, we found that the methylation level of LDHB was negatively correlated with the expression of multiple immune checkpoint proteins, and HCC patients with high methylation of LDHB respond better to immune checkpoint therapy. Recently, Kim et al. reported that cell-free DNA (cfDNA) methylation of LDHB can be used in the diagnosis of HCC [45]. Therefore, it is important to explore in-depth whether cfDNA methylation of LDHB alone or in combination with other cfDNA can be used as biomarkers for stratification of HCC immunotherapy.

There are several limitations in the current study. Although bioinformatics analysis demonstrated a remarkable relationship between LDHB mRNA levels and CD8^+^ T cell infiltration, it remains to be confirmed whether LDHB could recruit immune cells into TME in HCC clinical samples. While we found that LDHB suppressed HCC progress through modulating immune response, the detailed mechanism is still unclear. It is possible that LDHB may catalyze lactate oxidation to pyruvate, resulting in decreased lactate levels in intracellular and extracellular environments. On the one hand, Zhang et al. recently reported that lactate was an epigenetic regulator for the modulation of histones, termed as lactylation modification and thus modulating specific gene transcription [46]. Changes in lactate levels may affect histone lactylation modification in HCC cells, and thus alter the expression of certain chemokines or inflammatory factors. Indeed, Pan et al. found that H3 histone lactylation is elevated in liver cancer [47]. Alternatively, the release of lactate into the extracellular environment by cancer cells may not only increase acidification in the TME and promote immune escape [48], but might also be sensed by multiple types of immune cells, resulting in intracellular signaling and alter its functions in the TME [13]. Therefore, future studies are warranted to investigate whether LDHB regulates immune response through lactate levels and glycolysis.

In summary, this study identified a novel mechanism by which LDHB suppressed HCC progression via DNMT3A-induced hypermethylation of LDHB promoter and remodeling tumor immune environment. Our findings suggested that LDHB might become a promising prognostic biomarker and a novel target in HCC immunotherapy.

### Supplementary Information

Below is the link to the electronic supplementary material.

Supplementary file1 (DOCX 273 KB)

## Data Availability

Datasets containing expression, DNA methylation and clinical data were accessed from the TCGA-LIHC cohort and GEO website under the accession codes GSE63898 and GSE10141. Other data generated or analyzed during this study were included in this published article.
