# Supplementary material for: Epigenetic silencing of LDHB promotes hepatocellular carcinoma by remodeling the tumor microenvironment
Source: Cancer Immunol Immunother. 2024 May 13;73(7):127. doi: 10.1007/s00262-024-03717-2 (PMC11091036; doi:10.1007/s00262-024-03717-2)
Supplement: Supplementary file 1 — Supplementary file1 (DOCX 273 KB) [file 262_2024_3717_MOESM1_ESM.docx]

**Supplementary Figure 1**


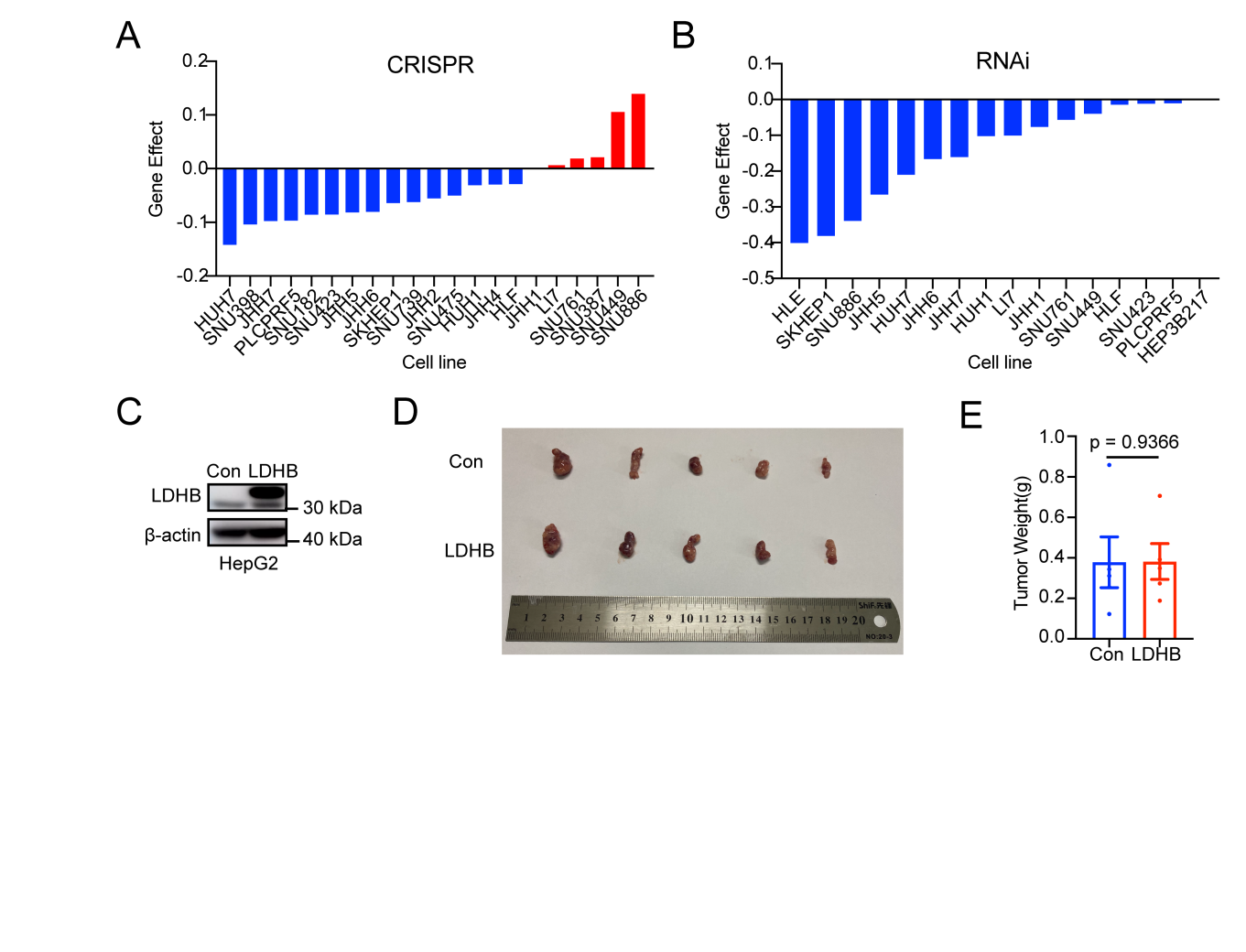


**Supplementary Figure 1.**  **LDHB suppresses HCC progression in immune-competent mice but not in immune-deficient mice.** (A, B) Barplots showing the LDHB essentiality in Liver cell lines from publicly available CRISPR-Cas9 screening (A) and RNAi screening (B). (C) LDHB expression in Hep G2/LDHB and corresponding control cells was examined by Western blotting. (D) HepG2 cells tested in C were inoculated into nude mice (n=5). Xenograft tumors at the endpoint were collected and displayed. (E) The weight of tumors was measured at the endpoint (D).
